# Comparative genomic and functional analyses of four sequenced *Bacillus cereus* genomes reveal conservation of genes relevant to plant-growth-promoting traits

**DOI:** 10.1038/s41598-018-35300-y

**Published:** 2018-11-19

**Authors:** Qingchao Zeng, Jianbo Xie, Yan Li, Tantan Gao, Cheng Xu, Qi Wang

**Affiliations:** 1 Key Laboratory of Plant Pathology, Ministry of Agriculture, College of Plant Protection, China Agricultural University, Beijing, 100193 P. R. China; 2 Key Laboratory of Genetics and Breeding in Forest Trees and Ornamental Plants, Ministry of Education, College of Biological Sciences and Technology, Beijing Forestry University, Beijing, 100083 P. R. China

## Abstract

Some *Bacillus* strains function as predominant plant-growth-promoting rhizobacteria. *Bacillus cereus* 905 is a rod-shaped Gram-positive bacterium isolated from wheat rhizosphere and is a rhizobacterium that exhibits significant plant-growth-promoting effects. Species belonging to the genus *Bacillus* are observed in numerous different habitats. Several papers on *B*. *cereus* are related to pathogens that causes food-borne illness and industrial applications. However, genomic analysis of plant-associated *B*. *cereus* has yet to be reported. Here, we conducted a genomic analysis comparing strain 905 with three other *B*. *cereus* strains and investigate the genomic characteristics and evolution traits of the species in different niches. The genome sizes of four *B*. *cereus* strains range from 5.38 M to 6.40 M, and the number of protein-coding genes varies in the four strains. Comparisons of the four *B*. *cereus* strains reveal 3,998 core genes. The function of strain-specific genes are related to carbohydrate, amino acid and coenzyme metabolism and transcription. Analysis of single nucleotide polymorphisms (SNPs) indicates local diversification of the four strains. SNPs are unevenly distributed throughout the four genomes, and function interpretation of regions with high SNP density coincides with the function of strain-specific genes. Detailed analysis indicates that certain SNPs contribute to the formation of strain-specific genes. By contrast, genes related to plant-growth-promoting traits are highly conserved. This study shows the genomic differences between four strains from different niches and provides an in-depth understanding of the genome architecture of these species, thus facilitating genetic engineering and agricultural applications in the future.

## Introduction

Plant-growth-promoting rhizobacteria (PGPR) are plant-associated, beneficial soil-dwelling organisms that competitively colonize the rhizosphere, rhizoplane, or roots. PGPR increases the fitness and growth of host plants through various mechanisms, including biological nitrogen fixation, phosphate solubilization, iron (Fe) acquisition by using siderophores and synthesis of plant hormones^1^. Owing to these good characteristics, biocontrol formulations using PGPR are increasingly applied in sustainable agriculture. The commercially utilized PGPR strains include species of *Agrobacterium*, *Azospirillum*, *Azotobacter*, *Bacillus*, *Burkholderia*, *Delfitia*, *Paenibacillus macerans*, *Pantoea agglomerans*, *Pseudomonas*, *Rhizobium* and *Serratia*^2^. The use of beneficial bacteria to combat pests or plant diseases has rapidly grown over the last decades. Given the negative impact of agrochemicals on the environment, the use of beneficial bacteria has been proven to be an efficient and environmental-friendly alternative to partially replace chemical pesticides. Among the different microbial species examined, members of the *Bacillus* genus are potential candidates for use as biological control agents^3^. *Bacillus* is a genus of Gram-positive, rod-shaped bacteria and is a member of the phylum Firmicutes. Several inoculants prepared from endospore-forming *Bacillus* strains are preferred, because their long-term viability facilitates the development of commercial products. For instance, *Bacillus amyloliquefaciens* FZB42 is commercially used as biocontrol bacterium owing to its efficiency against fungal and bacterial pathogens^4^. Members of the *Bacillus* genus are bacteria that exploit a wide of organic and inorganic substrates as nutrient sources^5^. Their production of antimicrobial substances and sporulation capacity provide *Bacillus* strains a dual advantage in terms of their survival in different habitats. Therefore, members of this genus are observed in various environments, such as soil, rhizosphere, plant, and sea water^5^. Given the wide spectrum of ecological, metabolic, and biochemical characteristics of the genus *Bacillus*, it is not surprising that diversity among *Bacillus* spp. extends to the genomic sequence level.

The *Bacillus cereus* group consists of Gram-positive, rod-shaped, spore-forming aerobic or facultatively anaerobic bacteria that are widespread in natural environments. Thus far, this group consists of 11 closely related species, that is, *B*. *anthracis*, *B*. *cereus*, *B*. *thuringiensis*, *B*. *mycoides*, *B*. *pseudomycoides*, *B*. *weihenstephanensis*, *B*. *cytotoxicus*, *B*. *toyonensis*, *B*. *gaemokensis*, *B*. *manliponensis* and *B*. *bingmayongensis*^6^. However, this group of strains has been notably resistant to any type of satisfactory classification. The *B*. *cereus* group comprises a highly versatile group of bacteria, which are of particular interest because of their ecological diversity ranging from saprophytic lifestyle in soil to symbiotic lifestyles near plant roots and in the guts of insects to various pathogenic lifestyles in diverse insect and mammalian hosts^7^. Importantly, *B*. *cereus* can contaminate food and cause emetic and diarrheal foodborne illnesses. The members of this group can be divided into pathogens and environmental strains of medical, industrial, and ecological relevance. Previous documents reported that bacteria of the *B*. *cereus* group produce various valuable enzymes and metabolites, degrade different types of pollutants, and promote the growth of animals and plants when used as probiotics^8^. However, a comprehensive analysis of the characteristics of *B*. *cereus* related to agriculture by using a comparative genomics approach has yet to be reported.

*B*. *cereus* 905, which is isolated from wheat rhizosphere, can colonize wheat rhizosphere with a large population size^9^. Meanwhile, this strain exhibits significant plant-growth-promoting effects. Thus, one commercial product developed from strain 905 has been applied to approximately 3 million acres of wheat fields after being registered as a biopesticide^10^. However, knowledge on the mechanism of the plant-growth-promoting activity of *B*. *cereus* 905 remains largely unknown. We sequenced the 905 genome to improve our understanding of the relevant *B*. *cereus* 905 strain. The genome sequences of certain *B*. *cereus* strains have been made available in the past several years. We conducted a detailed comparative genomic analysis of *B*. *cereus* 905 with three other plant-associated *B*. *cereus* strains exhibiting biocontrol activities and isolated from various plant habitats, including haw, alfalfa, and forest to enhance our understanding of the genomic differences between *B*. *cereus* strains isolated from different niches^11–13^. The genomes of each strain we are screened for the presence of loci associated with plant growth promotion. Specifically, we determined that certain single nucleotide polymorphisms (SNPs) contributed to the formation of strain-specific genes. Comparative genomic analysis provided information on the genetic basis of adaptation. This work offers a foundation for follow-up studies of target genes and functions and facilitate genetic engineering of *B*. *cereus* to improve agricultural and industrial applications.

## Results

### General genomic features

The assembly genome of *B*. *cereus* 905 produced 126 contigs with an *N*_50_ of 91,494 bp and the longest sequence of 307,306 bp. The genome size of strain 905 was 5,386,583 bp with a GC content of 35.04% (Table 1 and Fig. S1). The genome was predicted to possess a minimum of 5,492 protein-coding genes, 6 rRNA genes and 84 tRNA genes. The average length of the protein-coding genes was 822 bp, accounting for 83.80% of the genome sequence. Among the protein-coding genes, 3,802 genes can be assigned a putative function, whereas 1,690 genes were predicted to encode hypothetical proteins. In the database, the number of sequenced *B*. *cereus* strains that is increasing, providing the fundamental material for comparative genomic analysis. A summary of the features of each of the four genomes is shown in Table S1. The selected genomes presented a wide range of genome sizes from 5.39 Mb to 6.40 Mb, with the number of protein-coding genes ranging from 5,492 to 6,257, indicating substantial strain-to-strain variation (Table 1). Among the investigated *B*. *cereus* strains, *B*. *cereus* AR156 showed the highest GC content (35.25%), and the remaining *B*. *cereus* strains presented lower GC contents (34.81% to 35.04%).

**Table 1:** General genomic features of the four investigated *Bacillus cereus* strains.

	905	LCR12	UW85	AR156
Location of isolation	Wheat rhizosphere	Haw rhizosphere	Alfalfa roots	Forest soil
Chromosomes	126	40	54	1
Plasmid				3
Estimated genome size (bp)	5386583	6031538	6404077	5671798
GC content(%)	35.04	34.82	34.81	35.25
Protein-coding genes	5492	5911	6257	5725
rRNA genes	6	16	34	39
tRNA genes	84	86	50	99
NCBI Accession No.	LSTW00000000	MCAX00000000	LYVD00000000	CP015589

### Phylogenetic analysis

**Figure 1:**
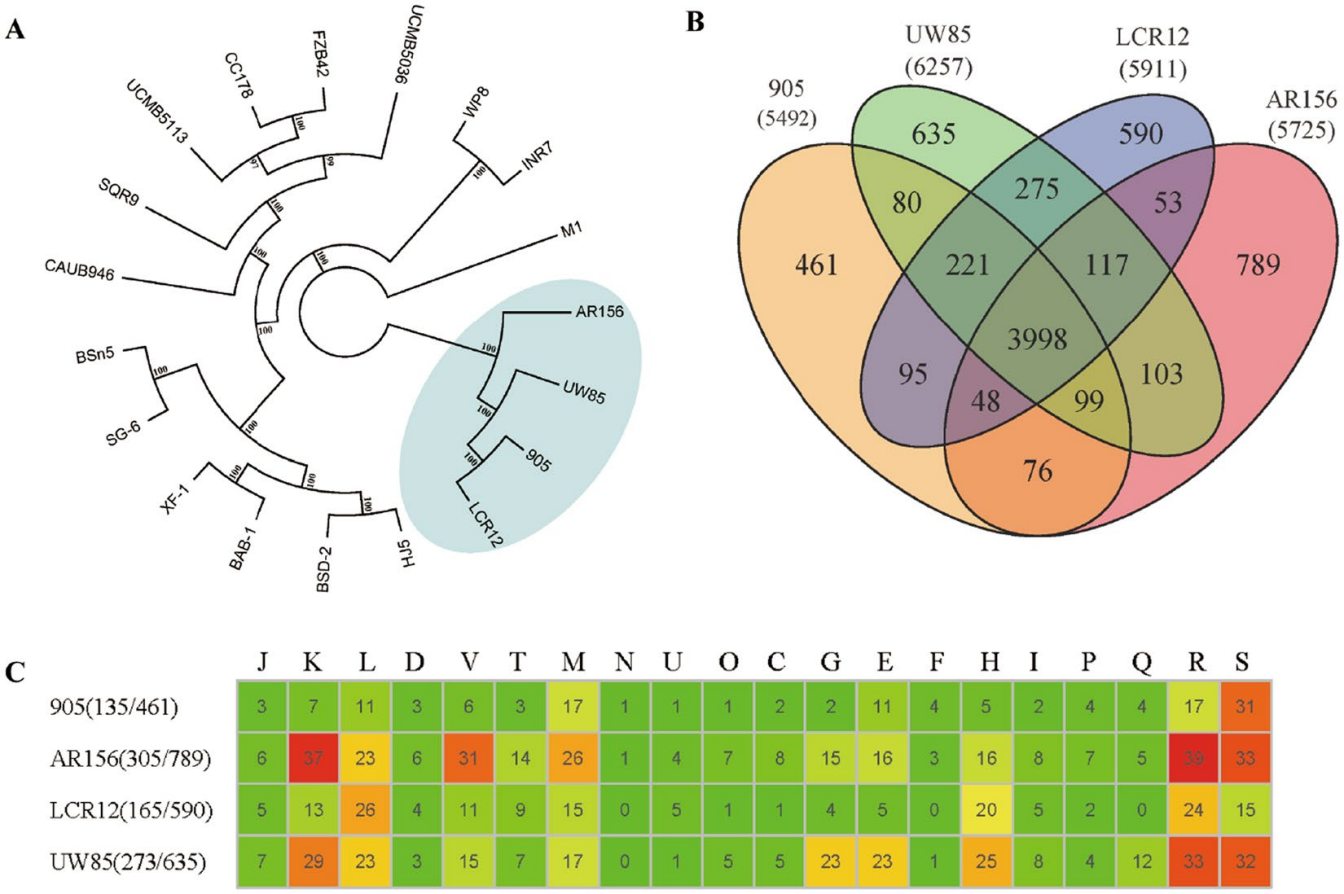
(**A**) Phylogenetic tree showing the relationship of the sequenced *Bacillus* spp. strains. The *Bacillus* strains included six *B*. *subtilis* (BSn5, SG-6, XF-1, BAB-1, BSD-2, and HJ5) and six *B*. *amyloliquefaciens* strains (CAUB946, SQR9, UCMB5113, CC178, FZB42, and UCMB5036) and two *B*. *pumilus* strains (WP8 and INR7) and four *B*. *cereus* strains(905, AR156, UW85, and LCR12). The tree is based on the 607 single-copy core genes and was generated using RAxML 8.2.10. *P*. *polymyxa* M1 was used as the out-group. Percent bootstrap values (from 100 replicates) are indicated at the nodes. (**B**) Genomic diversity of *B*. *cereus* strains. Each strain is represented by an oval. The number of orthologous protein-coding genes shared by all strains (the core genome) is at the center. Overlapping regions show the number of CDS represented by several strains. Numbers in nonoverlapping portions of each oval show the number of CDS unique to each strain. The total number of protein-coding genes within each genome is listed below the strain name. (**C**) Function classification of strain-specific genes in the four *B*. *cereus* strains. Comparison was based on 22 COGs categories: energy production and conversion (**C**); cell cycle control, cell division, and chromosome partitioning (**D**); amino acid transport and metabolism (**E**); nucleotide transport and metabolism (**F**); carbohydrate transport and metabolism (**G**); coenzyme transport and metabolism (**H**); lipid transport and metabolism (**I**); translation, ribosomal structure, and biogenesis (**J**); transcription (**K**); replication, recombination, and repair (L); cell wall, membrane, and envelope biogenesis (**M**); cell motility (**N**); posttranslational modification, protein turnover, and chaperones (**O**); inorganic transport and metabolism (**P**); secondary metabolites biosynthesis, transport, and catabolism (**Q**); general function prediction only (**R**); function unknown (**S**); signal transduction mechanisms (**T**); intracellular trafficking, secretion, and vesicular transport (**U**); and defense mechanisms (**V**).

The phylogenetic relationship of *B*. *cereus* 905 with other *Bacillus* spp strains was further analyzed. A total of 3,998 core genes, including 607 single-copy core genes, were identified by comparison of 18 *Bacillus* genomes and *Paenibacillus polymyxa* M1. The phylogenetic tree of the 18 *Bacillus* strains (four *B*. *cereus*, two *B*. *pumilus*, six *B*. *amyloliquefaciens*, and six *B*. *subtilis*) genomes was constructed based on the concatenation of the 607 single-copy core genes that were present in single copy in all genomes with Maximum likelihood (ML) method (Fig. 1A) and rooted by *P*. *polymyxa* M1. The phylogenetic trees, inferred with the Bayesian inference (BI) and Neighbour-joining (NJ) methods (Figs S2 and S3), are congruent with the ML phylogenetic tree. The phylogenetic tree shows that the selected *Bacillus* strains grouped into two clades. The *B*. *subtilis*, *B*. *amyloliquefaciens* and *B*. *pumilus* grouped into a single large clade composed of two subclades, whereas the remaining strains were allocated into another clade. In the large clade, *B*. *amyloliquefaciens* and *B*. *subtilis* formed a subclade, indicating that the two species are closely related, which is consistent with previous results^14^. The two strains of *B*. *pumilus* were allocated into another subclade (Fig. 1A). This analysis also showed a distant phylogenetic relationship between *B*. *cereus* and the other *Bacillus* strains.

When multiple genomes for the same species were available, the phylogeny results corresponded well with the species assignments based on average nucleotide identity (ANI) by using MUMmer (ANIm). Meanwhile, ANI calculated using MUMmer algorithm values provides a numerical and stable species boundary^15^. The strains *B*. *cereus* 905, UW85, and LCR12 show a high ANIm (>96%; Table S1). Values exceeding 96% are determined for strains of the same species^16^. However, it is noteworthy that strain AR156 should not be categorized as *B*. *cereus*, because its values are lower than 93% in the comparison of the genome of strain AR156 with those of *B*. *cereus* strains (905, UW85, and LCR12). We tested whether the phylogeny derived from the orthologous genes can reflect the phylogeny of the *Bacillus* strains. We also conducted hierarchal clustering analysis based on gene presence/absence among each strain, which also clearly distinguished the *B*. *cereus* strains and other *Bacillus* strains (Fig. S4), only with some discrepancies.

### Comparison among *B*. *cereus* genomes

Orthologous genes are clusters of genes in different species that have evolved by vertical descent from a single ancestral gene. A genome-wide comparison of orthologous clusters in different strains provides insight into the gene structure, gene function, and molecular evolution of genomes^17^. In total, 5,492, 5,725, 5,911, and 6,257 protein-coding genes (including hypothetical proteins) of the *B*. *cereus* strains 905, AR156, LCR12, and UW85, respectively, were annotated. The predicted proteins were functionally categorized using the Clusters of Orthologous Groups (COG) database. The COG categories were compared among the genomes and showed similar distributions among the four strains (Table S2). The total pan-genome for the four compared *B*. *cereus* strains encompasses 7,640 protein-coding genes. Among the 7,640 protein-coding genes, 3,998 genes, which accounted for 52.3% of the genes in the pan-genome of *B*. *cereus*, were represented in all genomes. We determined that the four strains shared a large set of core genome, which represent 63.9% to 72.8% of the repertoire of protein-coding genes, indicating a high similarity between these strains. Results confirmed those obtained from the Venn diagram, where the *B*. *cereus* strains are similar with a number of genes involved in different functions. The number of strain-specific genes ranges from 461 to 789, with the smallest gene identified in *B*. *cereus* 905 and the largest gene identified in *B*. *cereus* AR156 (Fig. 1B).

To examine the biological functions of unique genomes, we further used COG assignments to determine which functional category the strain-specific genes are classified. The percentage of genes assigned to each COG is represented in Fig. 1C. Although a large number of strain-specific genes (>60%) were not assigned to the COG categories, the remaining strain-specific genes fall into different functional categories. As shown in Fig. 1C, a high proportion of strain-specific genes in most of the strains were assigned to the E (Amino acid transport and metabolism), K (Transcription), L (Replication, recombination, and repair), G (Carbohydrate transport and metabolism), H (Coenzyme transport and metabolism), and M (Cell wall/membrane/envelope biogenesis) categories. These strains were probably isolated from different plants. Strain-specific metabolism in the adaption of each strain to a specific niche is important.

### Plant growth promotion

The mechanisms of PGPR can be divided into direct and indirect forms. Direct mechanisms comprise either the facilitation of resource acquisition (such as nitrogen fixation, phosphate solubilisation or Fe acquisition using siderophores) or the modulation of plant hormone levels (such as the production of indole-3-acetic acid (IAA) and employing the enzyme ACC deaminase)^1^. Meanwhile, indirect mechanisms aim to reduce of the inhibitory effects of pathogens on plant growth and development.

### IAA production

**Figure 2:**
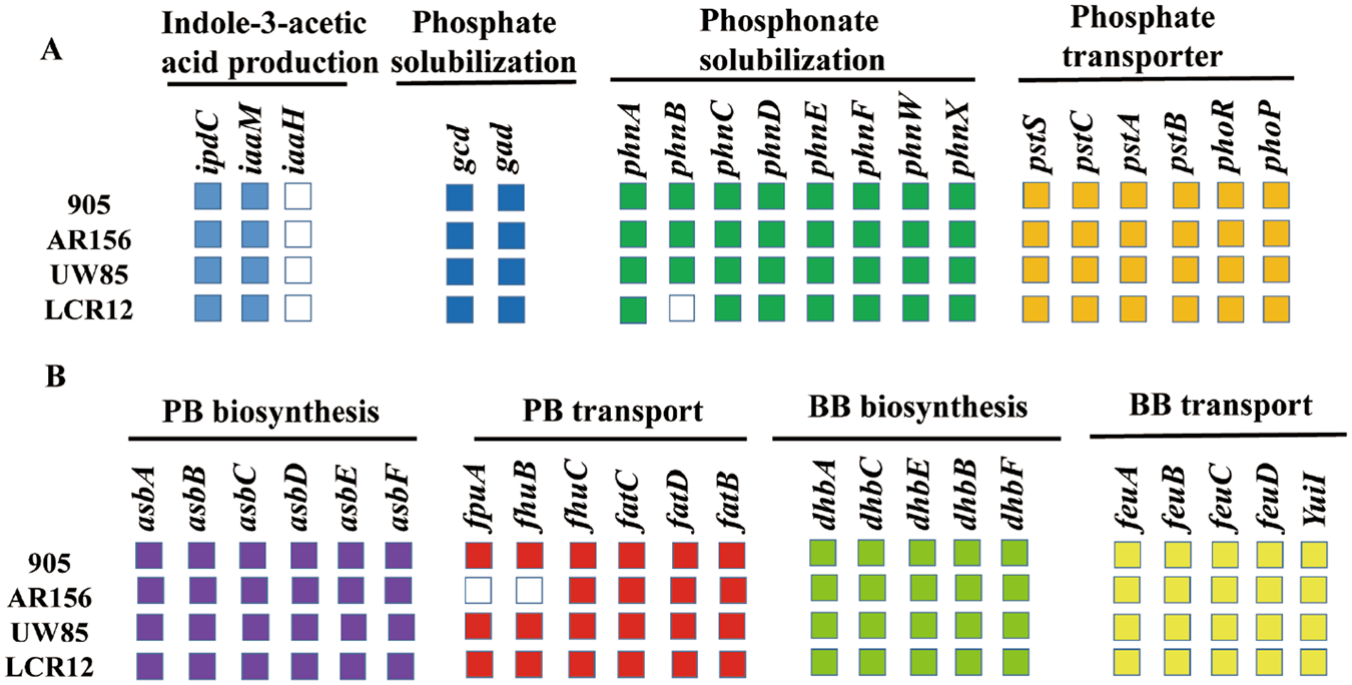
Plant-growth-promoting traits of the four *B*. *cereus* strains. (**A**) Genes involved in IAA production and organic and inorganic phosphate solubilization. (**B**) Genes involved in the production and transportation of siderophore. The colored box represents the presence of a gene within a genome, and the white box indicates the absence of a gene within a genome.

IAA plays an important role in the plant-growth-promoting effect of some plant-beneficial bacteria. Auxins produced by plant-associated bacteria typically induce root branching and elongation in plant host. We compared the four *B*. *cereus* genomes to determine the pathways involved in the production of IAA. In plants, four tryptophan-dependent IAA synthesis pathways, namely, indole-3-pyruvic acid (IPyA), indole-3-acetamide (IAM), indole-3-acetonitrile (IAN), tryptamine (TAM) pathways, have been postulated in bacteria^18,19^. The IAN pathway has not been characterized in bacteria, and the steps leading to the conversation of tryptophan to IAN are still a matter of debate. Meanwhile, for the biosynthesis of IAA via the TAM pathway, the genes involved in this pathway still need to be confirmed in bacteria^20^. In this study, we focused on the IPyA and IAM pathways. In the IPyA pathway, the *ipdC* gene, which encodes the key enzyme, has been identified in these four *B*. *cereus* strains. However, for other *Bacillus* strains, the well-documented indolepyruvate decarboxylase *ipdC* gene was undetected in the *B*. *amyloliquefaciens* SQR9^20^. The IAM pathway, which is catalyzed by the enzymes tryptophan-2-monooxygenase (IaaM) and IAM hydrolase (IaaH) is a two-step pathway. However, genes encoding IAM hydrolase were not detected in the four genomes (Fig. 2). This result showed that the IPyA pathway may be the main route for IAA production in the four *B*. *cereus* strains.

### Phosphorus solubilization and assimilation

Phosphorus (P) is one of the essential elements in plant development and growth^21^. However, a large portion of inorganic phosphates applied to the soil function as a fertilizer, are rapidly immobilized after application, and become unavailable to plants^22^. Some micro-organisms are capable of solubilizing insoluble P and mineralizing soil P for plant growth. Among all mechanisms, the solubilization of mineral phosphates by bacteria is typically achieved through gluconic acid production^21^. The production of gluconic acid was facilitated by glucose-1-dehydrogenase (*gcd*) and gluconic acid dehydrogenase (*gad*)^23^. In the research, we screened the two genes in *B*. *cereus* genomes and observed that all genomes contained both *gcd* and *gad* genes (Fig. 2A). This finding indicated that these *B*. *cereus* strains have the ability to solubilize inorganic mineral phosphates and are potential candidates as inoculants to increase P uptake by plants.

Another rich source of soil phosphate is trapped in the form of phosphonate, an organophosphorus compound that must be degraded prior to biological incorporation. The phosphonate gene cluster (*phn*) is responsible for bacterial degradation of phosphonates, which releases biologically available phosphate for nearby plants. The cluster of *phn* contains a 12.6-kb operon of 17 genes named, in alphabetical order, *phnA* to *phnQ*, in *Escherichia coli*^24,25^. All *B*. *cereus* strains do not carry the complete *phn* cluster and lack the genes encoding a C-P lyase protein (*phnGHIJKLMP*) responsible for phosphonate degradation into phosphate and an alkane. All strains appear to possess the capability to degrade phosphonoacetaldehyde (*phnX*) and phosphonoacetate (*phnA*). Among all the strain, only strain LCR12 did not contain *phnB* (Fig. 2A). These genome variations can be attributed to gene gain and loss events during the evolutionary process.

Phosphate-specific transporter (Pst) is a member of the ATP-binding cassette (ABC) family of permeases^25^, which is a major Pi transport system. Pst has been detected in bacteria, such as *B*. *subtilis*^26^, *E*. *coli*^27^, and *Mycobacterium tuberculosis*^28^. The *pst* genes in *E*. *coli* form an operon arranged in the order *pstS*, *pstC*, *pstA*, and *pstB*. However, there are two genes homologous to *pstB* in *B*. *subtilis*^25^. PhoP-PhoR, a two-component signal-transduction system, controls the expression of phosphonate uptake and C-P lyase activity in response to phosphate deficiency^29^. From the comparative genomics analysis, we determined that these four strains contained the *pstSCAB* operon and phoP-phoR system (Fig. 2A).

### Iron acquisition

Similar to P, Fe is abundant in soil and is mainly in a non-bioavailable form. Fe acts as a co-factor and electron acceptor in various essential enzymes and proteins and is an important nutrient for organisms^30^. Iron forms largely insoluble Fe^3+^ oxy-hydroxides, which are not readily used by either microorganisms or plants^31^. Under Fe limited conditions, most microorganisms can produce siderophores, which are extracellular, low molecular weight Fe^3+^ chelators. The soluble Fe^3+^-siderophore complexes are available to plants and microorganisms. Members of the *B*. *cereus* sensu lato group are known to produce and utilize two siderophores, namely, bacillibactin (BB) and petrobactin (PB)^33^. Siderophores are synthesized mainly by nonribosomal peptide synthetases, which are encoded by gene clusters^31^. The cluster for the synthesis of bacillibactin is composed of *dhbACBEF* in *B*. *cereus*, whereas the biosynthesis genes of petrobactin is composed of *asbABCDEF*^33^. Our comparative genomics analysis revealed that the four *B*. *cereus* strains carry the *dhb* cluster (*dhbACBEF*). Petrobactin biosynthesis genes (*asbABCDEF*) are also present in all *B*. *cereus* strains (Fig. 2B).

Fe, an essential element for bacterial growth, reportedly affects biofilm formation^34^. However, biofilm formation may serve as a survival mechanism in different environments and function as an important factor contributing to host colonization. Thus, we focused on transporters of siderophore in the *B*. *cereus* strain. Siderophore uptake in Gram-positive bacteria is facilitated by membrane-bound substrate-binding proteins and membrane-spanning ABC transporters^35,36^. The uptake of bacillibactin in members of the *B*. *cereus* sensu lato group possibly involves *feuABCD* and *yuiI*^32^. The above genes were detected in the four *B*. *cereus* strains. Meanwhile, the gene *ymfD*, which exports bacillibactin, was identified in these four strains. For petrobactin, the uptake of this siderophore originated from the *fpuA/fhuB* and *fatBCD/fhuC* gene clusters^32^. In addition to strain AR156, the other strains possess all of these genes. The *fpuA* and *fhuB* were undetected in strain AR156 (Fig. 2B). This result showed that these biocontrol strains may exhibit the capability to obtain Fe and colonized plants.

### Local diversification of *B*. *cereus* strains

**Figure 3:**
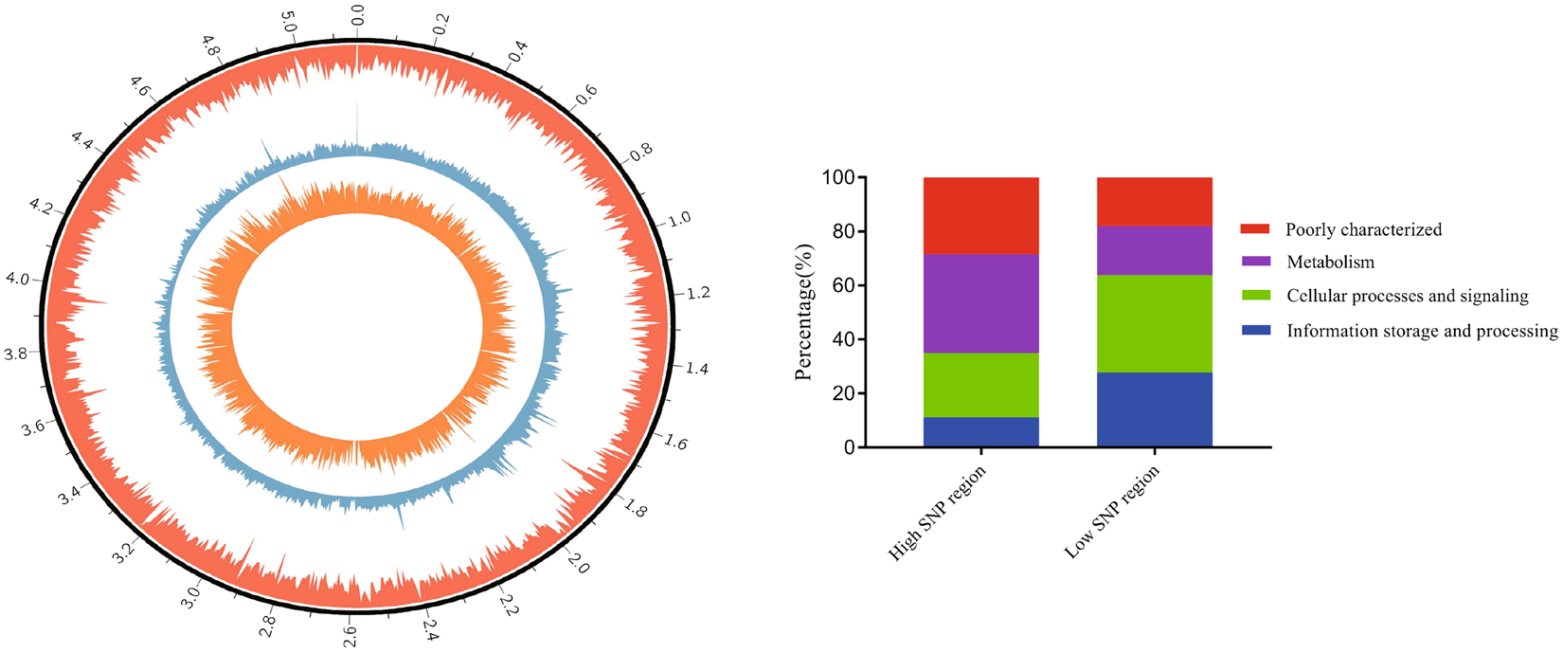
(**A**) Chromosome map and distribution of SNP in the four *B*. *cereus* strains. From the outside of the circle to the inside of the circle: Circle 1 is the gene density map (red). Circle 2 is the GC content density map (green). Circle 3 is the distribution of SNP (orange). (**B**) Relative proportions (%) of high-SNP and low-SNP region CDSs in each COG super-functional category.

Genetic variation is a prerequisite for biological evolution^37^. Some of these factors leave an imprint on sequence variation across the entire genome. Most of the DNA variations detected in bacterial species are in the form of SNPs^38^. Thus, SNPs are ideal tools to investigate the genomic imprint of adaption to different niches^29^. We used Mummer to align the contigs from the three strains (905, LCR12, and UW85) to strain AR156 to analyze the patterns of SNPs distribution. Our analysis produced a total of 1033 SNPs regions throughout the genomes by using a sliding windows of 5 kb. We also determined that the SNPs were not evenly distributed among these regions (Fig. 3A). Among these regions, 17 regions showed almost no SNPs and 21 regions contained more than 140 SNPs/kb. Then, we compared the high and low SNP regions by using COG assignments to determine whether there were differences in the proportion of the high SNP regions that may be attributed to a particular cellular process. High SNP regions were found to be disproportionally enriched in metabolism (Fisher’s exact test; p-value < 0.05). Similarly, low SNP regions were enriched in information storage and processing (Fisher’s exact test; p-value < 0.05) compared with high SNP regions (Fig. 3B; Table S3). PGPR produces substances affecting plant growth and development and PGPR consumes carbohydrates and amino acids released by the plant^39^. Differences in the types and contents of root exudates from different tree species were reported^40^. In this study, the four *B*. *cereus* strains were isolated from various plants. According to detailed analysis and with certain genes as example, *aroK* (shikimate kinase), *hisH* (imidazole glycerol phosphate synthase), *ppsA* (phosphoenolpyruvate synthase), and *eamA* (amino-acid metabolite efflux pump) were related to the high SNPs regions. Meanwhile, the genes were also annotated to carbohydrate and amino acid transport and metabolism in the COG database, possibly implying that the high SNP regions are related to metabolism. Previous studies reported that the evolution of glycometabolism is a key factor in the environmental adaptability of the genus *Paenibacillus*^41^.

**Figure 4:**
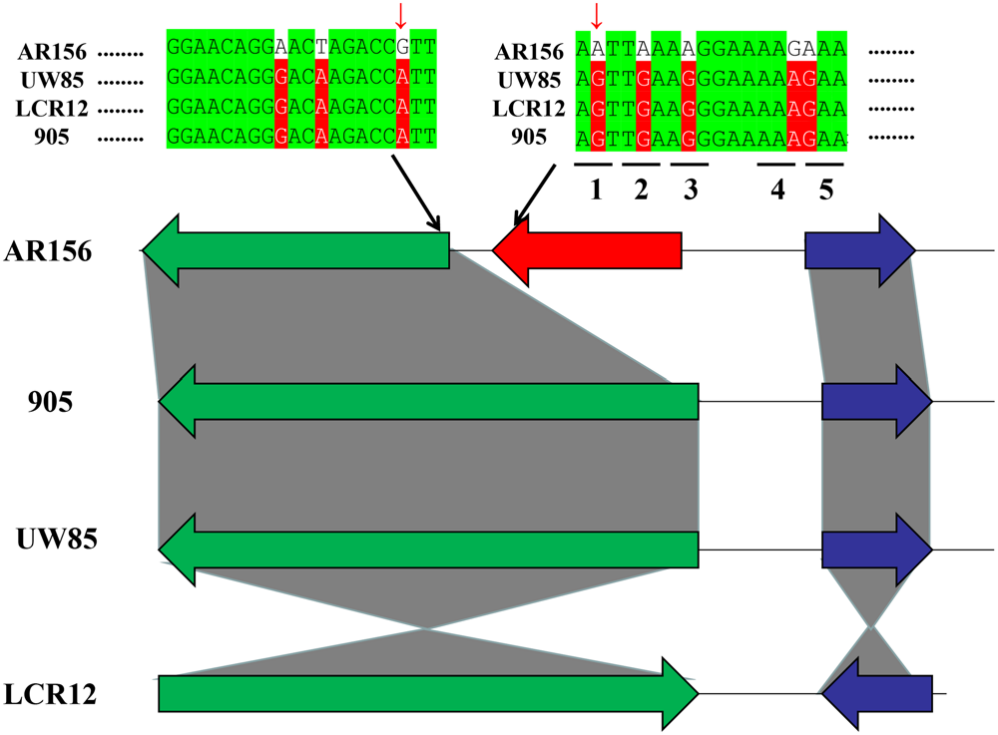
SNP related to the strain-specific gene. The red gene represents the specific gene. In the genome sequence, the green nucleotide represents the conserved region, and the red nucleotide represents the SNP regions. The red arrow indicates the SNPs in the initiation codon and stop codons. The numbers 1–5 represented the codon in genome sequence.

Point mutations are often assumed to be the raw material of evolution^42^. Synonymous mutation is defined that in a DNA sequence that encodes a protein but produces no change in the amino acid and nonsynonymous mutation, resulting in an amino acid change that may influence the function of the encoded protein. In the study, several SNPs were synonymous mutations (Fig. 4, eg. 1). Moreover, nonsynonymous mutations were detected in the genome sequences (Fig. 4, egs 2–5). Furthermore, for AR156, we compared the strain-specific genes with genes related to high SNP regions. This result showed that a part of the strain-specific genes were present in the high SNPs regions. Then, we focused on the same genes for further analysis. Figure 4 shows that putative penicillin-binding protein PbpX which is a specific gene for AR156 was detected SNP in the stop codon and that additional SNPs were nonsynonymous mutations in the genome sequence. Compared with other strains, these SNPs in AR156 possibly contributed to the formation of specific genes. However, single nucleotide insertion or deletion in protein coding sequences results in a frameshift such as the downstream codons being translated from a different reading frame, leading to significant alteration of the encoded protein^42^. For strain 905, one gene contained certain SNPs that cause frameshift mutation. This result indicated the formation of strain-specific genes from point mutations, particularly SNPs (Figure S5).

## Discussion

The genomic and evolutionary characteristics of *B*. *cereus*, particularly the root-colonizing strains, are currently garnering considerable interest because of their potential for use in agriculture. In this study, we conducted a comparative genomic analysis among the four *B*. *cereus* strains, which were previously sequenced. Phylogenetic trees, which were constructed based on 607 single copy core genes by using three different methods (NJ, ML, BI), show a high consistency with each other. The results showed that the four *B*. *cereus* strains (905, AR156, LCR12, and UW85) formed a clade, and the 14 other strains (six *B*. *amyloliquefaciens* strains, six *B*. *subtilis* strains and two *B*. *pumilus* strains) formed a large clade. The hierarchal clustering of orthologous genes based on the presence/absence matrix showed a similar pattern. Meantime, ANI analysis also supported the results. According to the calculated ANI values, the values in the *B*. *cereus* clade were lower than 93% when the genome of strain AR156 was compared with those of *B*. *cereus* strains (905, UW85, and LCR12). Strain AR156 may represent a species other than *B*. *cereus* and should not be named as *B*. *cereus* (ANIm < 93%). For the *B*. *pumilus*, we will select reference strain to calculate the ANI values in the future.

The genome size and GC content of microorganisms influence their extensive adaptabilities to the environment^43^. Under most conditions, strains with a larger genome size may be generally adaptive to complex habitats because these microorganisms may encode additional products for metabolism and stress tolerance^44^. However, several papers suggested that the small size of bacterial genomes may be also competitive, indicating advantages in energy saving and reproductive efficiency^45,46^. The results showed that the GC contents between the four *B*. *cereus* strains were different. Meanwhile, genome sizes vary from 5.39 M to 6.40 M. However, these *B*. *cereus* strains were all isolated from the soil. Genome size and GC content also differed. In the future, we will select more strains which isolated from different habitats (such as rhizosphere, freshwater, and marine water) and explore the genomic characteristics among the strains. Ecologically, the multiplicity of the rRNA operon is a potential mechanism for adaption to different environmental conditions^47^.

In *B*. *creus* 905 and LCR12, the number of rRNA genes was less than *B*. *cereus* AR156 and UW85. In general terms, bacteria that posses more rRNA operon copies may cope better with fluctuating nutrient inputs than bacteria with fewer rRNA operon copies, which tend to live in environments where nutrients are scarce^48^. However, the result shows that the rhizosphere strains have less rRNA operons. But the genome sequence was not complete, we need to further study about it.

The genus *Bacillus* exhibits extensive environmental adaptability and can populate various ecological niches. The pan-genome of the *B*. *cereus* is open^49^, indicating that *B*. *cereus* tends to acquire new genes to enhance the adaptability. Bacteria must change their genetic material to adapt to variable environmental conditions, thus a high niche diversity reflects larger pan-genomes^50,51^. Given the wide distribution of *B*. *cereus*, a large pan-genome size corresponds to diverse living conditions. To our knowledge, more studies were related to the pathogenicity of *B*. *cereus*. The *B*. *cereus* strains have more agricultural and industrial application. We should pay attention to more *B*. *cereus* strains, which are related to agriculture. To investigate the pan-genome of the *B*. *cereus* and explore the evolutionary reason for the wide niche adaptation of these bacteria are fundamental to learn more about this strain. Meanwhile, we could learn the evolution of the *B*. *cereus* strains. Comparative genome analysis of the genus *Novosphingobium* isolated from different environments was conducted. The results showed that habitat-specific genes and regulatory hubs that could determine habitat selection for *Novosphingobium* spp^52^. The pan-genome analysis is highly valuable to identify niche-specific genes and guide future studies linking genes to ecological niches. In the four *B*. *cereus* strains, the pan-genome includes 7,640 CDS, with 3,998 CDS is identified as core genomes. The gene repertoires of bacteria are dynamic and *B*. *cereus* abandoned some dispensable genes while acquiring new characteristics to adopt. Thus, we focused on the specific genes of the *B*. *cereus* strains. According to the analysis of the specific genes of the *B*. *cereus* strains, the results showed that the specific genes are related to amino acid, carbohydrate, and coenzyme transport and metabolism.

SNPs may fall within coding sequences of genes and non-coding regions of genes. If SNPs occur in non-coding regions, probably production a new protein coding genes comparing others strains. However, SNPs occur in the coding region which is a nonsynonymous mutation that could lead to amino acid change. The results showed that strain AR156 exhibited some SNPs related to the formation of strain-specific genes comparing the four strains (Fig. 4). Meanwhile, in strain 905, the SNPs in the genome sequence caused frameshift mutation. Some paper stated that some SNPs related to resistence to teicoplanin in *Staphylococcus aureus*^42^. We also learn that some SNPs related to adaption to different environment in the future.

In summary, through comparative genomic analysis of the four *B*. *cereus* strains, we present a global view of these genomes. However, a small difference in the plant-growth-promoting genes among the four *B*. *cereus* strains was observed. Despite their geographical isolation and varied plant hosts, the majority of genes implicated in plant association are highly conserved amongst *B*. *cereus* strains. In particular, genes responsible for IAA production, P solubilization and assimilation, and iron acquisition are largely conserved among these strains.

## Methods

### Growth conditions and genomic DNA preparation

The *B*. *cereus* 905 was initially grown at 30 °C or 37 °C in lysogenic broth (LB) (10 g tryptone, 5 g yeast extract, and 5 g NaCI per liter broth) broth or on solid LB medium supplemented with 1.5% agar. The total genomic DNA of *B*. *cereus* 905 was extracted by using Phenol-chloroform method^53^. After extraction, the quality of the DNA was measured using a NanoDrop spectrophotometer (Thermo Scientific NanoDrop 2000).

### Genome sequencing, assembly, and annotation

The genome sequence of the PGPR strain *B*. *cereus* 905 was already submitted to the National Center for Biotechnology Information (NCBI) database with the accession number of LSTW00000000.1. The *B*. *cereus* 905 genome was sequenced using the Genome Sequencer FLX platform (Roche, Mannheim, Germany)^10^. The library for strain 905 was constructed using a 3-kb-long paired-end tag with a GS FLX library preparation kit^10^. The output reads were assembled using the GS De Novo Assembler software program. Gene prediction of this PGPR strain was annotated by the Prokka^54^ software, which is a rapid prokaryotic genome annotation software. Annotation of the protein-coding sequence was conducted using the Basic Local Alignment Search Tool (BLAST) against the COG, Kyoto Encyclopedia of Genes and Genomes, NCBI nonredundant protein and Interpro databases. The genome sequence of *B*. *cereus* ATCC 10987 was set as the reference genome, and the contigs of *B*. *cereus* 905 was ordered by Mauve^55^. The final annotated chromosome was plotted using CIRCOS^56^ to show the gene locations, GC skew, and GC content.

### Genome comparisons

The genome sequence of *B*. *cereus* 905 was aligned against the sequences of other *B*. *cereus* genomes obtained from NCBI database. The accession numbers for the four *B*. *cereus* genomes used in the comparative analysis with *B*. *cereus* 905 were CP015589 (*B*. *cereus* AR156), MCAX00000000 (*B*. *cereus* LCR12), and LYVD00000000 (*B*. *cereus* UW85). PGAP pipeline^57^ based protein similarity method was used to detect a set of core orthologs from the four *B*. *cereus* strains and the core orthologs were clustered at least 50% protein sequence identity to each other and 50% overlap with the longest sequence with an *e*-value 1e-5. The dataset of shared genes among the four strains was defined as their core genomes, and the total set of genes within test genomes was defined as the pan-genome. Strain-specific genes were extracted from the orthologous table by using Perl script. Strain-specific genes were assigned by a BLAST search against the COG database, with an *e*-value of 1e-5. The ANI values between genomes were calculated by using the NUCmer algorithm integrated in Jspecies^15^ with default settings.

Protein homology was determined by performing a BLASTp search against identified homologs. Proteins with an *e*-value and positive amino acid identity cutoff of ≤10^−25^ and ≥60%, respectively, were considered homologous.

### Phylogenetic studies

A phylogenetic tree for the set of 19 genomes was inferred using a core genome alignment concatenation approach. Multiple alignments of amino acid sequences were conducted using MAFFT^58^ (version 7.310), and conserved blocks from multiple alignments of test protein were selected by using Gblocks^59^. Phylogenetic trees were constructed using three methods, namely, NJ, ML, and BI. Phylogenetic trees were inferred from 607 single-copy core genes shared by 19 strains, including an out-group (*P*. *polymyxa* M1). The ML tree was constructed using the RAxML (version 8.2.10) software and the PROTGAMMALGX model with 100 bootstrap replicates. A Bayesian phylogram was obtained using MrBayes 3.2.6. (http://mrbayes.sourceforge.net/) with 10,000 mcmc generations; every 10 trees were sampled and a consensus tree was obtained after the first 250 generations were removed using the burnin command. The NJ whole genome phylogeny was generated using the protdist and neighbor packages in PHYLIP 3.696. FigTree v1.4.3, Mega, or iTOL (http://itol.embl.de/) software was employed to show the trees.

To evaluate the phylogeny of the *Bacillus* strains, we constructed a 0/1 matrix, with 1 indicating the presence of orthologous genes in genome and 0 indicating the absence of that orthologous genes in genome. Hierarchical clustering was used to analyze the 0/1 matrix as implemented in the R package pvclust with 500 bootstrap replicates^60^.

### SNP mapping

The Mummer^61^ software was used to detect all SNPs, and all of the *B*. *cereus* genome sequences were mapped to the reference genome sequence of *B*. *cereus* AR156. From all SNPs identified in the four *B*. *cereus* genome sequences, the density of the SNP distribution was calculated throughout the reference *B*. *cereus* AR156 genome by using a sliding-window size of 5 kb (step of the sliding windows = 5 kb). In all of these regions, the top 3% regions were considered high SNP regions, whereas the last 3% regions were considered low SNP regions. The corresponding genes were extracted for subsequent analysis. Then, the genes were assigned to the COG database with an *e*-value of 1e-5.

## Electronic supplementary material

Supporting Information
