# Supplementary material for: Comparative genomic and functional analyses of four sequenced Bacillus cereus genomes reveal conservation of genes relevant to plant-growth-promoting traits
Source: Sci Rep. 2018 Nov 19;8:17009. doi: 10.1038/s41598-018-35300-y (PMC6242881; doi:10.1038/s41598-018-35300-y)
Supplement: Supplementary file 1 — Supporting Information [file 41598_2018_35300_MOESM1_ESM.pdf]

Comparative genomic and functional analyses of four sequenced *Bacillus cereus* genomes reveal conservation of genes relevant to plant-growth-promoting traits

Qingchao Zeng[1], Jianbo Xie[2], Yan Li[1], Tantan Gao[1], Cheng Xu[1] and Qi Wang[1,*]

[1]Key Laboratory of Plant Pathology, Ministry of Agriculture, College of Plant Protection, China Agricultural University, Beijing, 100193, P. R. China.

[2]Key Laboratory of Genetics and Breeding in Forest Trees and Ornamental Plants, Ministry of Education, College of Biological Sciences and Technology, Beijing Forestry University, Beijing, 100083, P. R. China.

Table S1 The average nucleotide identity (ANI) values amongst different strains of *Bacillus*. The ANI based on whole-genome comparison of strain 905 with those of other *Bacillu*s strains.

	BSD-2	905	WP8	UW85	CAU B946	FZB42	INR7	XF-1	AR156	CC178	SG-6	LCR12	BSn5	BAB-1	SQR9	UCMB5113	HJ5	UCMB5036
BSD-2	---																	
905	83.86	---																
WP8	84.9	85.57	---															
UW85	82.41	96.47	84.42	---														
CAU B946	84.48	86.18	85.57	87.44	---													
FZB42	84.52	85.57	85.66	87.95	97.76	---												
INR7	85.04	85.03	88.98	86.6	84.92	84.86	---											
XF-1	99.92	84.26	85.03	86.03	84.47	84.53	85.3	---										
AR156	83.51	92.22	85.39	92.14	84.71	84.68	85.88	83.43	---									
CC178	84.52	85.52	85.63	87.98	97.76	99.98	84.9	84.53	84.71	---								
SG-6	98.34	84.16	84.77	86.71	84.3	84.39	84.68	98.28	83.95	84.36	---							
LCR12	83.73	96.87	85.76	96.3	85.08	85.15	85.41	83.81	92.11	85.15	84.44	---						
BSn5	98.32	84	84.44	86.08	84.26	84.26	84.69	98.26	83.4	84.32	98.75	85.25	---					
BAB-1	99.95	83.69	84.73	85.68	84.07	84.32	84.94	99.9	83.44	84.28	98.33	84.94	98.29	---				
SQR9	84.42	86.48	85.64	88.14	97.68	98.48	84.73	84.62	84.89	98.48	84.28	86.62	84.44	84.26	---			
UCMB5113	84.52	86.43	85.66	88.09	97.83	98.87	84.95	84.51	84.95	98.88	84.37	86.35	84.29	84.3	98.46	---		
HJ5	99.98	83.79	85.02	85.4	84.29	84.38	85.22	99.92	83.42	84.37	98.34	84.95	98.32	99.95	84.5	84.36	---	
UCMB5036	84.57	86.72	85.48	88.58	97.79	98.87	84.82	84.7	84.65	98.86	84.36	86.13	84.4	84.34	98.33	99.32	84.44	---

Table S2 Comparative analysis of the functional categories based on COG of the protein-coding genes of the *B. cereus* strains analyzed.

**Functional categories based on COG (%)**	**905**	**AR156**	**LCR12**	**UW85**
[A]RNA processing and modification	1(0.03)	1(0.03)	0(0)	1(0.03)
[C]Energy production and conversion	202(5.40)	203(5.47)	203(5.20)	200(5.17)
[D]Cell cycle control, cell division, chromosome partitioning	42(1.12)	41(1.11)	44(1.13)	41(1.06)
[E]Amino acid transport and metabolism	361(9.66)	361(9.73)	368(9.42)	377(9.74)
[F]Nucleotide transport and metabolism	107(2.86)	103(2.78)	105(2.69)	107(2.77)
[G]Carbohydrate transport and metabolism	185(4.95)	188(5.07)	189(4.84)	201(5.20)
[H]Coenzyme transport and metabolism	252(6.74)	255(6.87)	270(6.91)	262(6.77)
[I]Lipid transport and metabolism	92(2.46)	99(2.67)	105(2.69)	96(2.48)
[J]Translation, ribosomal structure and biogenesis	212(5.67)	211(5.69)	212(5.43)	207(5.35)

[K]Transcription	282(7.54)	298(8.03)	289(7.40)	296(7.65)
[L]Replication, recombination and repair	149(3.99)	136(3.67)	182(4.66)	200(5.17)
[M]Cell wall/membrane/envelope biogenesis	239(6.39)	227(6.12)	262(6.71)	237(6.13)
[N]Cell motility	20(0.54)	18(0.49)	18(0.46)	18(0.47)
[O]Posttranslational modification, protein turnover, chaperones	128(3.42)	126(3.40)	131(3.35)	136(3.52)
[P]Inorganic ion transport and metabolism	207(5.54)	211(5.69)	216(5.53)	219(5.66)
[Q]Secondary metabolites biosynthesis, transport and catabolism	47(1.26)	39(1.05)	56(1.43)	55(1.42)
[R]General function prediction only	476(12.73)	468(12.61)	501(12.83)	485(12.54)
[S]Function unknown	467(12.49)	452(12.18)	463(11.85)	461(11.92)
[T]Signal transduction mechanisms	117(3.13)	123(3.32)	131(3.35)	121(3.13)
[U]Intracellular trafficking, secretion, and vesicular transport	33(0.88)	31(0.84)	38(0.97)	29(0.75)
[V]Defense mechanisms	119(3.18)	119(3.21)	123(3.15)	120(3.10)

Table S3 Comparison of the COG assignments between high and low SNP regions. The SNP distribution was calculated throughout the reference *B. cereus* AR156 genome by using a sliding-window size of 5 kb. The top 3% regions were considered high SNP regions, whereas the last 3% regions were considered low SNP regions.

	High SNP region	Low SNP region	*p*-value*
**Information storage and processing**	**14**	**20**	0.02165
Translation, ribosomal structure and biogenesis	2	14	
RNA processing and modification	0	0	
Transcription	9	3	
Replication, recombination and repair	3	3	
Chromatin structure and dynamics	0	0	
**Cellular processes and signaling**	**30**	**26**	0.2134
Cell cycle control, cell division, chromosome partitioning	0	1	

Nuclear structure	0	0	
Defense mechanisms	7	6	
Signal transduction mechanisms	7	2	
Cell wall/membrane/envelope biogenesis	6	12	
Cell motility	0	2	
Cytoskeleton	0	0	
Extracellular structures	0	0	
Intracellular trafficking, secretion, and vesicular transport	1	1	
Posttranslational modification, protein turnover, chaperones	9	2	
**Metabolism**	**46**	**13**	0.04172
Energy production and conversion	6	2	
Carbohydrate transport and metabolism	4	3	

Amino acid transport and metabolism	11	5	
Nucleotide transport and metabolism	4	0	
Coenzyme transport and metabolism	13	0	
Lipid transport and metabolism	2	1	
Inorganic ion transport and metabolism	6	2	
Secondary metabolites biosynthesis, transport and catabolism	0	0	
**Poorly characterized**	**36**	**13**	0.2404
General function prediction only	18	5	
Function unknown	18	8	
**Total**	**126**	**72**	

*:Fisher's exact test.

Figure S1 Genome map of *Bacillus cereus* 905. Each ring of the circle has a different genome information: rings from the outside in: (1) scale marks (unit: Mb), (2, 3) protein-coding genes on the forward and reverse strands, respectively (color-coded by the functional categories), (4, 5) rRNA (blue) and tRNA (red) on the forward and reverse strands, respectively, (6) GC content (positive: red; negative: blue), and (7) GC skew (above average: aquamarine; below average: orange).

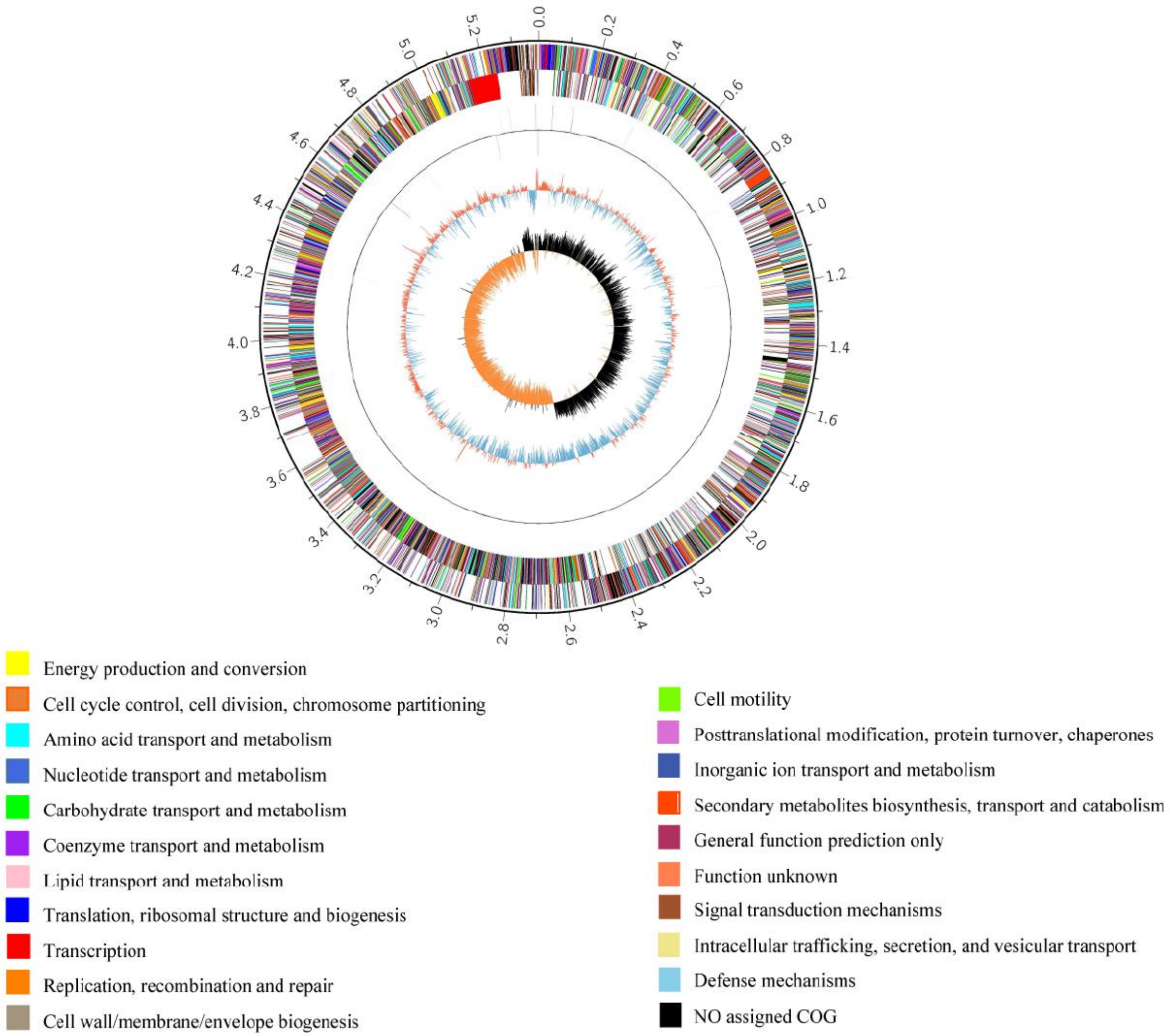
0.0
0.2
0.4
0.6
0.8
1.0
1.2
1.4
1.6
1.8
2.0
2.2
2.4
2.6
2.8
3.0
3.2
3.4
3.6
3.8
4.0
4.2
4.4
4.6
4.8
5.0
5.2
Energy production and conversion
Cell cycle control, cell division, chromosome partitioning
Amino acid transport and metabolism
Nucleotide transport and metabolism
Carbohydrate transport and metabolism
Coenzyme transport and metabolism
Lipid transport and metabolism
Translation, ribosomal structure and biogenesis
Transcription
Replication, recombination and repair
Cell wall/membrane/envelope biogenesis
Cell motility
Posttranslational modification, protein turnover, chaperones
Inorganic ion transport and metabolism
Secondary metabolites biosynthesis, transport and catabolism
General function prediction only
Function unknown
Signal transduction mechanisms
Intracellular trafficking, secretion, and vesicular transport
Defense mechanisms
NO assigned COG

Figure S2 Phylogenetic tree of 18 *Bacillus* genomes based on the Bayesian inference method. The phylogenetic tree was constructed using 607 single-copy core genes shared by 18 genomes and an out-group (*Paenibacillus polymyxa* M1). The phylogenetic tree was rooted by the out-group. Support values are shown for nodes as bayesian inference posterior probability model bootstrap. Branch lengths were estimated through Bayesian analysis, and the scale bar denotes substitutions per sites.

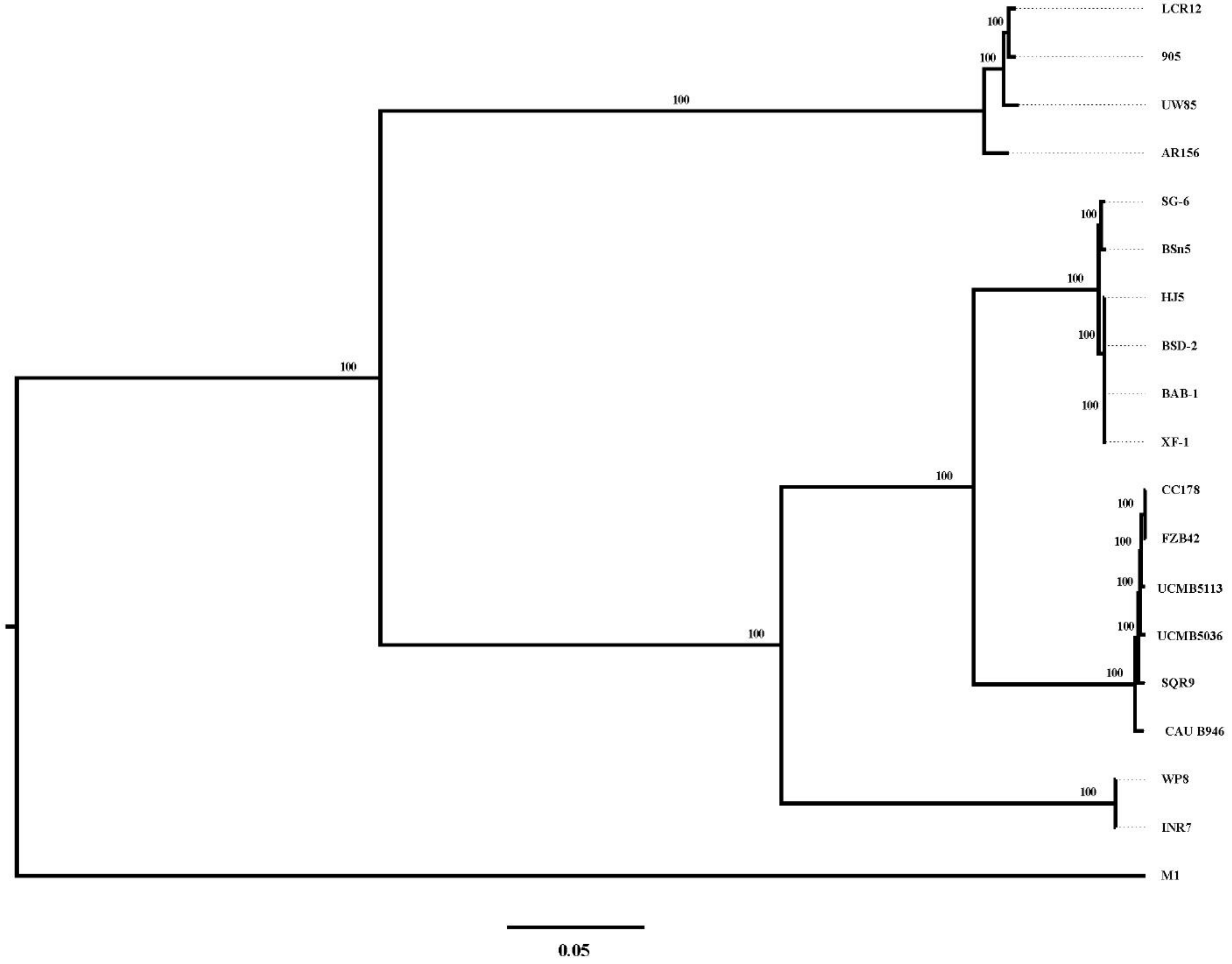
LCR12
905
UW85
AR156
SG-6
BSn5
HJ5
BSD-2
BAB-1
XF-1
CC178
FZB42
UCMB5113
UCMB5036
SQR9
CAU B946
WP8
INR7
M1
100
0.05

Figure S3 Phylogenetics tree of 18 *Bacillus* genomes based on the Neighbor-Joining method. The phylogenetic tree was constructed using 607 single-copy core genes shared by 18 genomes and an out-group (*P. polymyxa* M1). The phylogenetic tree was rooted by the out-group. Bootstrap values are shown for each node, and the scale bar represents the number of substitutions per site. Branch lengths are proportional to the amount of evolutionary change.

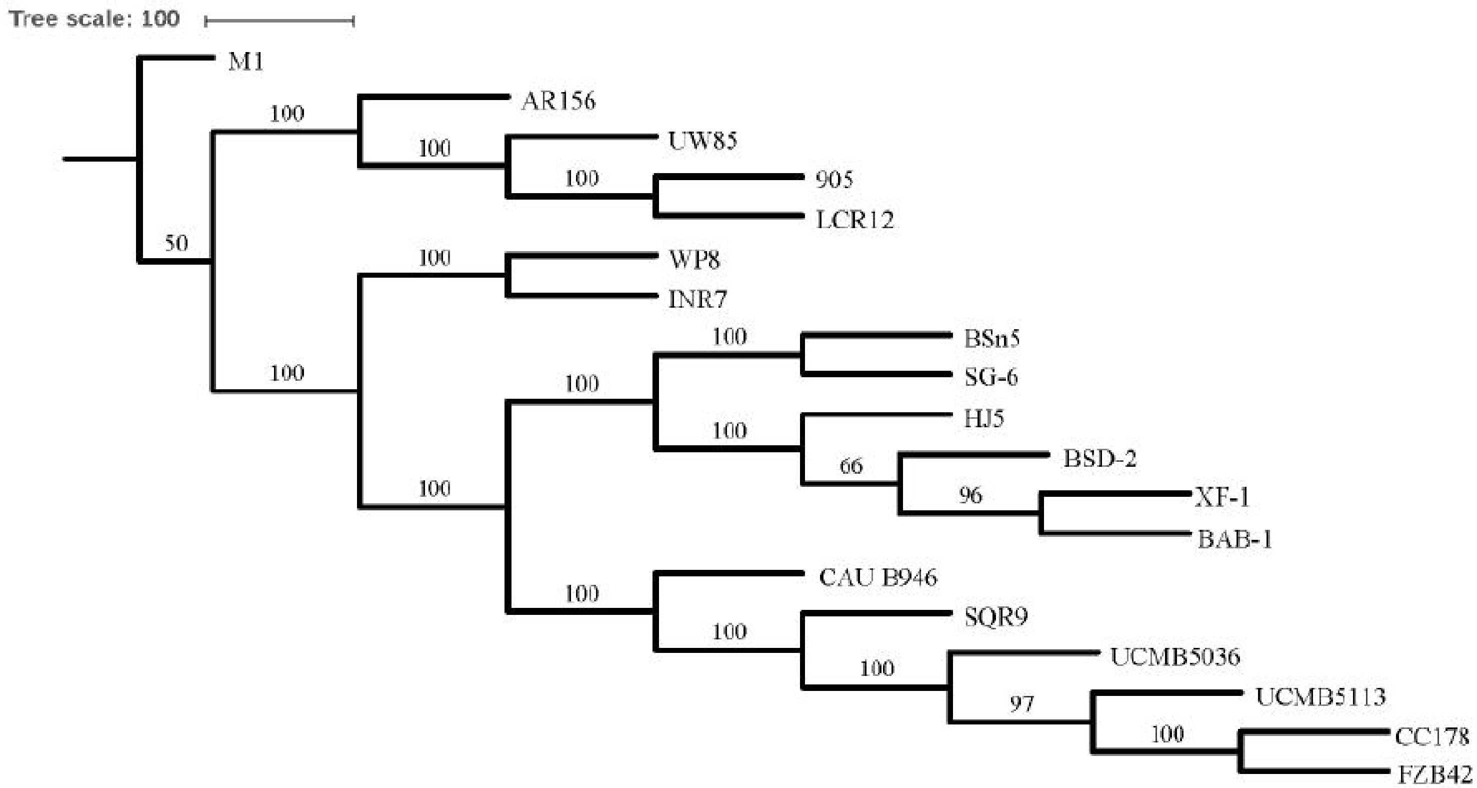
Tree scale: 100
M1
AR156
UW85
905
LCR12
WP8
INR7
BSn5
SG-6
HJ5
BSD-2
XF-1
BAB-1
CAU B946
SQR9
UCMB5036
UCMB5113
CC178
FZB42
100
100
100
50
100
100
100
100
100
66
96
100
100
100
97
100

Figure S4 Hierarchal clustering among genomes using the presence/absence of orthologous groups. Approximately unbiased probability and bootstrap probability (au/bp) values were indicated at each node.

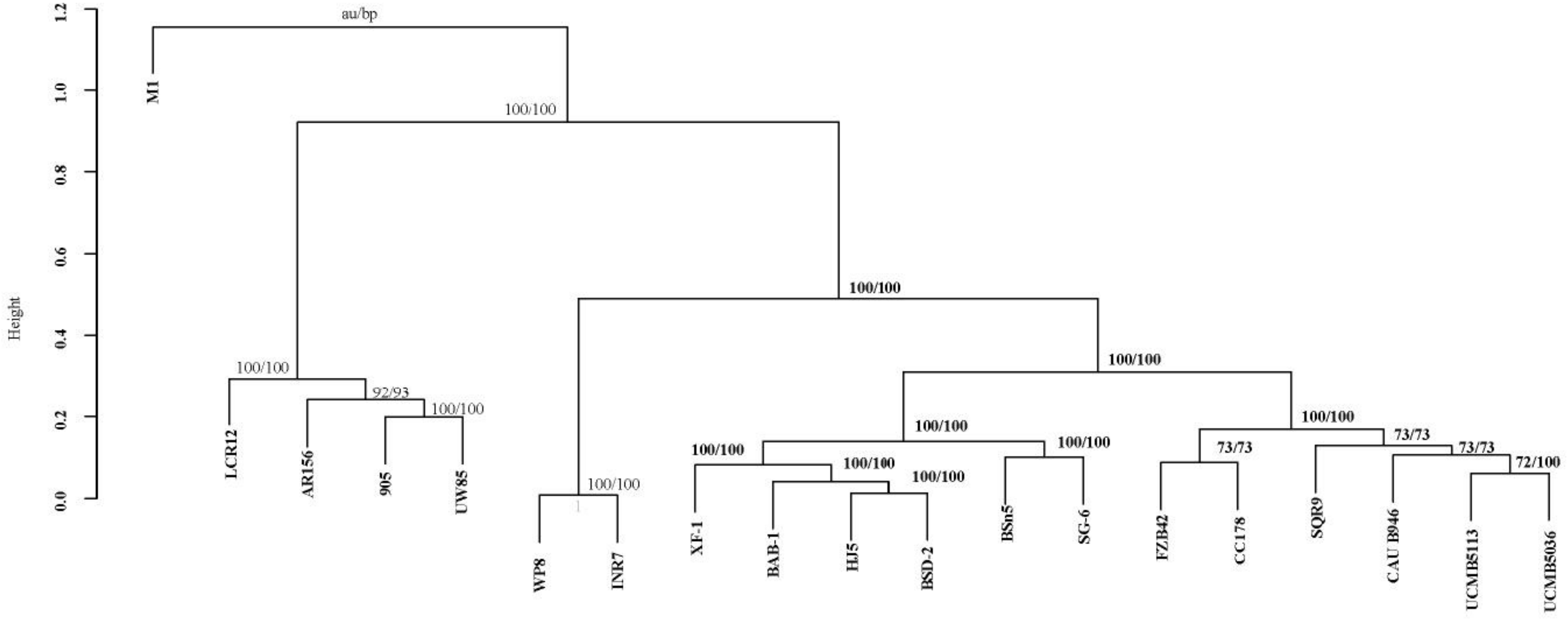

Figure S5 The SNPs and insertion nucleotides in the *B. cereus* 905 genome sequence. The red gene represents the strain-specific gene of *B. cereus* 905. In the genome sequence, the green nucleotide represents the conserved region, and the red nucleotide represents SNP regions. The red box indicates the insertion regions in the genome sequence.

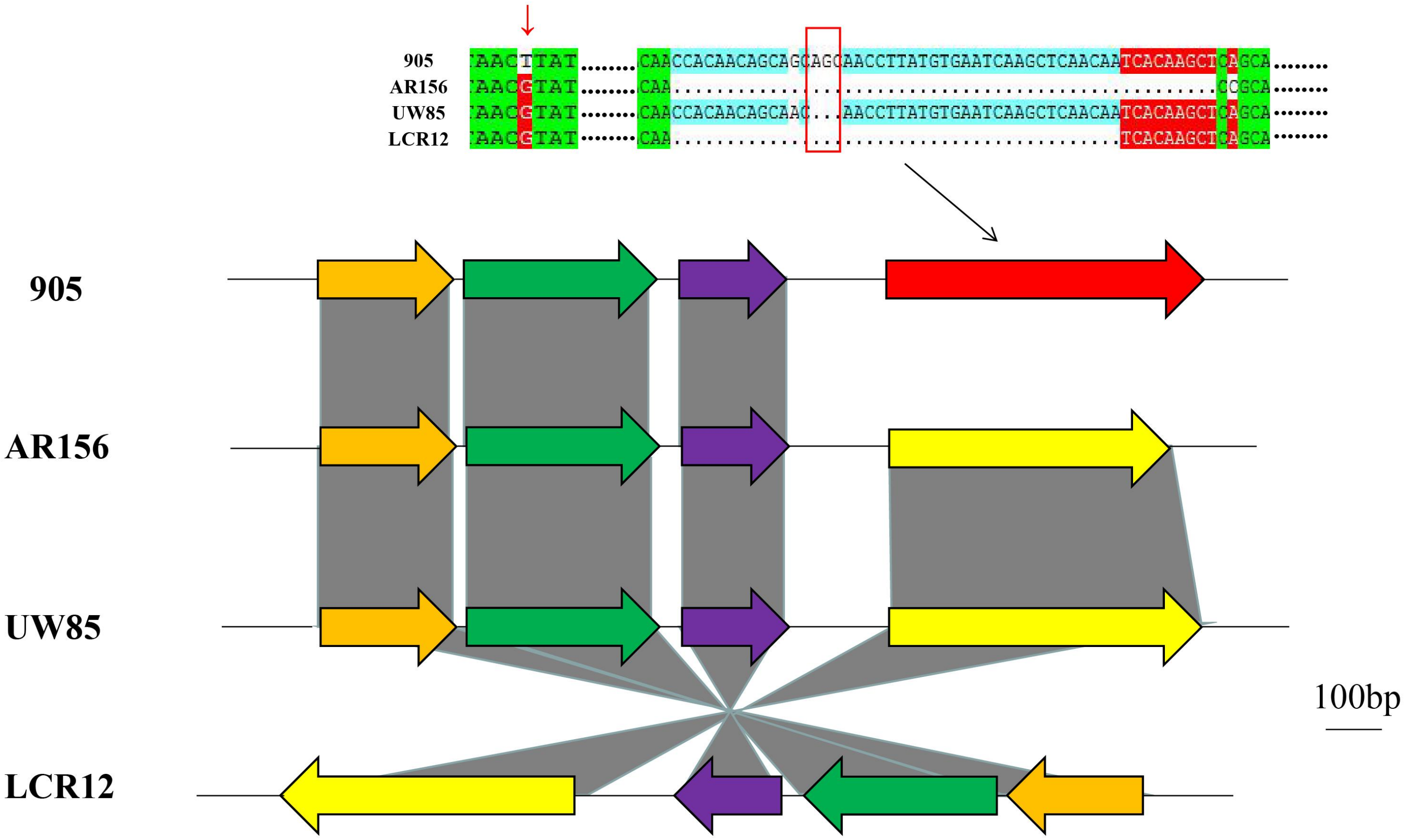
905 TAACTTAT........CAACCACAACAGCAGCAGCAACCTTATGTGAATCAAGCTCAACAATCACAAGCTCAGCA........
AR156 TAACGTAT........CAA..CCGCA........
UW85 TAACGTAT........CAACCACAACAGCAAC...AACCTTATGTGAATCAAGCTCAACAATCACAAGCTCAGCA........
LCR12 TAACGTAT........CAA...................................TCACAAGCTCAGCA........
905
AR156
UW85
LCR12
100bp
